# An Optimal Bahadur-Efficient Method in Detection of Sparse Signals with Applications to Pathway Analysis in Sequencing Association Studies

**DOI:** 10.1371/journal.pone.0152667

**Published:** 2016-07-05

**Authors:** Hongying Dai, Guodong Wu, Michael Wu, Degui Zhi

**Affiliations:** 1 Health Services and Outcomes Research, Children’s Mercy Hospital, Kansas City, MO, United States of America; 2 Department of Biomedical & Health Informatics, University of Missouri-Kansas City, Kansas City, MO, United States of America; 3 Lovelace Respiratory Research Institute, Albuquerque, New Mexico, United States of America; 4 Biostatistics and Biomathematics Program, Public Health Sciences Division, Fred Hutchinson Cancer Research Center, Seattle, WA, United States of America; 5 Department of Biostatistics, University of Alabama at Birmingham, Birmingham, AL, United States of America; New Jersey Institute of Technology, UNITED STATES

## Abstract

Next-generation sequencing data pose a severe curse of dimensionality, complicating traditional "single marker—single trait" analysis. We propose a two-stage combined p-value method for pathway analysis. The first stage is at the gene level, where we integrate effects within a gene using the Sequence Kernel Association Test (SKAT). The second stage is at the pathway level, where we perform a correlated Lancaster procedure to detect joint effects from multiple genes within a pathway. We show that the Lancaster procedure is optimal in Bahadur efficiency among all combined p-value methods. The Bahadur efficiency,$\underset{\varepsilon \to 0}{\text{lim}}N^{\left(2\right)}/N^{\left(1\right)}=\phi_{12}(\theta )$, compares sample sizes among different statistical tests when signals become sparse in sequencing data, i.e. *ε* →0. The optimal Bahadur efficiency ensures that the Lancaster procedure asymptotically requires a minimal sample size to detect sparse signals ($P_{N^{\left(i\right)}}<\varepsilon \to 0$). The Lancaster procedure can also be applied to meta-analysis. Extensive empirical assessments of exome sequencing data show that the proposed method outperforms Gene Set Enrichment Analysis (GSEA). We applied the competitive Lancaster procedure to meta-analysis data generated by the Global Lipids Genetics Consortium to identify pathways significantly associated with high-density lipoprotein cholesterol, low-density lipoprotein cholesterol, triglycerides, and total cholesterol.

## Introduction

Next-generation sequencing (NGS) technology has opened a new era for studying genetic associations with complex diseases. Yet, although whole genome searching has become easier and less costly to perform, our ability to critically evaluate such high throughput data has not improved substantially. Sequencing data often contain millions of genetic variants. However, testing millions of markers using the "single marker—single trait" analysis often loses power after the multiple-testing adjustment. Genome-wide significance requires strict Bonferroni correction with p-value < 2.5×10^−6^ for a total of 20,000 gene-based statistical tests. To maintain statistical power of detecting rare variants, a theoretical sample size of n>10,000 may be required for sequencing data [1].

These dimensional challenges motivate us to aggregate effects from multiple genes using pathway analysis. Genetic pathways comprise molecular entities that interact with each other to regulate specific cell functions, metabolic processes, biosynthesis, and embryonic developments. For non-Mendelian diseases and complex traits, multiple genetic risk factors may function together in the pathway. As a result, signals may not be significant in the "single marker-single trait" analysis, but many such values from related genes might provide valuable information regarding gene function and regulation. The pathway information can be extracted from bioinformatic resources, such as the Kyoto Encyclopedia of Genes and Genomes (KEGG) [2], the PANTHER classification system for protein sequence data [3], and Reactome database for human pathway data [4].

We propose a two-stage combined p-value method for pathway (gene set) analysis of NGS data. The first stage is at the gene level, where we integrate effects from rare and common variants within a gene. The goal of the first stage analysis is to generate a p-value that summarizes an overall effect within a gene. The second stage is at the pathway level, where we aggregate p-values among all genes in a pathway.

An exome sequencing simulation study was conducted to compare the SKAT-Lancaster procedure and Gene Set Enrichment Analysis (GSEA) [5]. We applied the competitive Lancaster procedure to meta-analysis data generated by the Global Lipids Genetics Consortium.

## Methods

### Two-Stage Pathway Analysis for Sequencing Data

There is a different nature of effects between gene and pathway. At the gene level, we are interested in identifying *rare* genetic variants from high throughput data. At the pathway level, genes with similar functions work together to fulfill biological tasks. Thus, we are interested in detecting *small and common* effects among genes. The proposed “SKAT-Lancaster” procedure provides a two-stage framework in order to (1) reduce the dimension of genetic variants, (2) combine effects from multiple genes, and (3) take genetic correlation architecture into account.

#### Stage I—Gene Level Testing

In the first stage, we suggest integrating effects from rare variants within the *i*^*th*^ gene using the Sequence Kernel Association Test (SKAT) [6]. Several tests have been proposed to analyze rare variants at the gene level, including burden tests and the C-alpha test. We choose SKAT because SKAT has been proven to be a locally most powerful score test [7].

Let *G*_*ij*_ be the *j*^*th*^ variant of the *i*^*th*^ gene. Let *β*_*i*_ = (*β*_*i*1_, ⋯, *β*_*ij*_, ⋯) be the effects from markers in the *i*^*th*^ gene. Generate a p-value, *P*_*i*_ for the *i*^*th*^ hypothesis testing $H_{0i}:\beta_{i}\;=\vec{0}$ vs. $H_{ai}:\beta_{i}\neq \vec{0}$ in the *i*^*th*^ gene. A → is added to denote the zero vector. SKAT is a locally most powerful score test on the variance component of a regression model $Y=\alpha^{t}X+\beta_{i}^{t}G_{i}+\varepsilon$, where *Y* is a phenotype, *α* is a vector of fixed effects from covariates *X*, and *ε* is an error term. To increase the power, SKAT tests *H*_0*i*_: *β*_*i*_ = 0 by treating *β*_*ij*_ as a random variable with mean zero and variance *w*_*ij*_*τ*_*i*_, where *τ*_*i*_ is a common variance component and *w*_*ij*_ is a pre-specified weight for variant *G*_*ij*_. As a result,$H_{0i}:\beta_{i}=\vec{0}$ is equivalent to *H*_0*i*_: *τ*_*i*_ = 0. The variance component score statistic is $Q={\left(Y-\hat{\mu }\right)}^{t}G_{i}W_{i}G_{i}^{t}(Y-\hat{\mu })$, where $\hat{\mu }={\hat{\alpha }}^{t}X$ is the predicted mean of *Y* under *H*_0*i*_, and *W*_*i*_ = *diag*(*w*_*i*1_, ⋯) are the weights of the variants. Under the null hypothesis, *Q* follows a mixture of chi-square distributions [6].

Common variants, population stratification, and other covariates can also be included as fixed effects in the model. The goal of the first stage analysis is to generate a p-value that summarizes the overall effect for each gene.

#### Stage II—Pathway Level P-value Combination

The second stage is at the pathway level, where we perform the modified Lancaster procedure to combine effects from multiple genes within a pathway. We choose the Lancaster procedure because it is optimal in Bahadur efficiency among all weighted combined p-value methods. The original Lancaster procedure is based on the independent p-value assumption. However, genetic data are highly correlated and ignoring the correlation structure will severely inflate the Type I error rate. Thus we need a modification of the Lancaster procedure to take the complex correlation structure among genetic variants into account [8].

Consider *m* sequences of test statistics, $\{T_{n_{i}}^{\left(i\right)}\},\;i=1,\;2,\;\cdots ,\;m$ and the corresponding significance levels,$\left\{P_{n_{i}}^{\left(i\right)}\right\}$, where *n*_*i*_ is the sample size for the *i*^*th*^ test statistic. Let the Lancaster statistic $T_{n}^{Lancaster}=\sum_{i=1}^{m}F_{i}^{-1}(1-P_{n_{i}}^{\left(i\right)})$, where $P_{n_{i}}^{\left(i\right)}$ $F_{i}^{-1}$ is the inverse cumulative distribution function (CDF) of $\chi_{w_{i}}^{2}$ with *w*_*i*_ > 0 for *i* = 1, 2,⋯, *m*. When p-values are correlated, $T_{n}^{Lancaster}$ does not follow $\chi_{\sum_{i=1}^{m}w_{i}}^{2}$. The null distribution of $T_{n}^{Lancaster}$ does not have an explicit analytical form. To address this issue, we approximate the $T_{n}^{Lancaster}$ statistic with a scaled chi-square distribution. Let $T_{n}^{Lancaster}\approx c\chi_{v}^{2}$ where *c* > 0 is a scalar and *v* > 0 is the degrees of freedom for the approximate chi-square distribution. Under *H*_0_: *θ* ∈ Θ_0_, we have

$$E(T_{n}^{Lancaster})=E\left(\sum_{i=1}^{m}F_{i}^{-1}(1-P_{n_{i}}^{\left(i\right)})\right)=\sum_{i=1}^{m}w_{i}$$

and

$$\text{var}(T_{n}^{Lancaster})=\sum_{i=1}^{m}\text{var}\left(F_{i}^{-1}(1-P_{n_{i}}^{\left(i\right)})\right)+2\sum_{i<j}\text{cov}\left(F_{i}^{-1}(1-P_{n_{i}}^{\left(i\right)}),F_{j}^{-1}(1-P_{n_{j}}^{\left(j\right)})\right)=2\sum_{i=1}^{n}w_{i}+2\sum_{i<j}\rho_{ij},$$

where $\rho_{ij}=\text{cov}\left(F_{i}^{-1}(1-P_{n_{i}}^{\left(i\right)}),F_{j}^{-1}(1-P_{n_{j}}^{\left(j\right)})\right)$ takes the correlation among p-values into account. We use the Satterthwaite method to match the mean and variance of $T_{n}^{Lancaster}$ and $c\chi_{v}^{2}$, and solve the equations to derive *c* and *v*. Thus we have $T_{n}^{Lancaster}\approx c\chi_{v}^{2}$, where $c=0.5\text{var}(T_{n}^{Lancaster})/E(T_{n}^{Lancaster})$ and $v=2{\left[E(T_{n}^{Lancaster})\right]}^{2}/\text{var}(T_{n}^{Lancaster})$.

As genetic variants have very complex correlation architecture, there is no analytical form for the exact correlated Lancaster procedure. The Satterthwaite approximation is an effective approach to summarize the distribution of the exact correlated Lancaster procedure. Q-Q plots from simulated data suggest a good match between the approximated $T_{n}^{Lancaster}$ and exact $T_{n}^{Lancaster}$, with a very slight deviation in the tail part. By introducing the correlation structure, the Satterthwaite approximation can significantly reduce the Type I error among correlated p-values.

### Self Contained vs. Competitive Lancaster Procedure

The main difference between competitive and self-contained tests lies in the formulation of the null hypothesis [9]. Let *μ*_*i*_ stand for the effect size from the *i*^*th*^ pathway. The null hypothesis for the self-contained test of the *i*^*th*^ pathway is H_0, self-contained_: *μ*_*i*_ = 0. Thus, the correlated Lancaster procedure can be considered as a self-contained test.

The null hypothesis in the competitive test is H_0, competitive_: *μ*_1_ = *μ*_2_ = ⋯ = *μ*_*i*_ = ⋯ The competitive Lancaster test can be carried out using permutation testing:

Step 1: Let *P*_*i*_ be the p-value from the Lancaster procedure in the *i*^*th*^ real pathway.
Step 2: Create *L*, say 100000, permutated pathways by shuffling genes among pathways. The permutated pathway sizes should resemble the real pathway sizes. Let *P*^*l*^ be the p-value from the Lancaster procedure in the *l*^*th*^ permutated pathway for *l* = 1, 2,⋯, *L*.
Step 3: The p-value of the competitive Lancaster procedure is $\sum_{l=1}^{L}I\{P_{i}\geq P^{l}\}/L$ for the *i*^*th*^ real pathway, where *I*{.} is an indicator function.

### Meta-analysis in Sequencing Association Studies

Due to cost, the rarity of diseases involved, and high dimensionality of variants, sequencing association studies are often underpowered to detect modest genetic effects. Meta-analysis can be used to address this issue by analyzing data across studies. Meta-analysis uses study-specific summary statistics, allowing investigators to combine information across studies when individual-level data cannot be shared.

The Lancaster procedure is independent from the SKAT test. One can directly apply the Lancaster procedure to meta-analysis, as we demonstrated in our analysis of the Global Lipids Genetics Consortium data. In this work, we choose SKAT to pair with the Lancaster procedure in order to detect rare variants in exome sequencing data. For other types of sequencing data, we suggest replacing SKAT with other statistical tests, such as FaST-LMM [10] or GEMMA [11], at the gene level and then applying the Lancaster procedure to combine multiple effects at the pathway level.

### Lancaster Procedure Is Optimal in Bahadur Efficiency

Several weighted combined p-value methods have been developed. See [12] for a comprehensive review. Since high throughput sequencing data pose a severe challenge in retaining the statistical power for small sample sizes in detection of sparse signals, it is critical to theoretically assess the efficiency among the weighted combined p-value methods. Let *P*_*i*_, (*i* = 1, 2,⋯, *m*) be p-values from *m* hypothesis tests. Littell and Folks [13, 14] showed that Fisher's method of combining independent tests ($T^{Fisher}=-2\sum_{i=1}^{m}\text{ln}(P_{i})$) is asymptotically Bahadur efficient. However, Fisher's method does not allow a weight function when combining p-values.

The weight function can be used to integrate multiple-source omics data from varying sequencing platforms. For instance, one can apply weight functions to integrate microarray data and CHIP-TIE data to identify the protein involved in transcription. In this case, weight functions can be considered as prior information to ensure the binding calling is a real signal instead of an artifact. As [15] pointed out, carefully chosen weights can generally improve power for a combination of p-values.

There is no uniformly most powerful method of combining p-values. The Bahadur efficiency is an important way to compare sample sizes required by two statistics in detection of sparse signals (*ε* → 0).

#### The Notation of Bahadur Relative Efficiency

Consider a hypothesis test for *H*_0_: *θ* ∈ Θ_0_ vs. *H*_*a*_: *θ* ∈ Θ − Θ_0_. Bahadur efficiency offers an asymptotic relative comparison between two competing test statistics. Under *H*_*a*_, a test statistic whose significance level converges to zero at a faster rate is considered more Bahadur efficient.

Let *T*_*n*_ be a real valued test statistic depending on an independent sample, *x*_1_, *x*_2_,⋯, *x*_*n*_ for *n* = 1, 2, ⋯ Assume for all *θ* ∈ Θ_0_, *T*_*n*_ follows the same null CDF *F*_0_. Let *t* be the value attained by *T*_*n*_, then the significance level of *T*_*n*_ is *P*_*n*_ = 1 − *F*_0_(*t*). Suppose that − 2ln *P*_*n*_ / *n* converges to *c*(*θ*) with probability 1, i.e.,

$$\text{Pr}\left(\underset{n\to \infty }{\text{lim}}-(2/n)\text{ln}P_{n}=c(\theta )\right)=1$$

for some *c*(*θ*) > 0 under *H*_*a*_: *θ* ∈ Θ−Θ_0_. The value *c*(*θ*) is dependent on *θ* under the alternative hypothesis and *c*(*θ*) is called the Bahadur efficiency slope of *T*_*n*_ when *n* → *∞*. Consider two competing sequences of test statistics, $\left\{T_{n}{}^{\left(1\right)}\right\}$ and $\left\{T_{n}{}^{\left(2\right)}\right\}$, with the Bahadur efficiency slopes *c*_1_(*θ*) and *c*_2_(*θ*), respectively. The ratio

$$\phi_{12}(\theta )=c_{1}(\theta )/c_{2}(\theta )$$

is the Bahadur efficiency of $\left\{T_{n}{}^{\left(1\right)}\right\}$ relative to $\left\{T_{n}{}^{\left(2\right)}\right\}$. Let *N*^(*i*)^ be the minimal sample size satisfying $P_{N^{\left(i\right)}}<\varepsilon$ for the *i*^*th*^ test. Bahadur [16] shows that

$$\underset{\varepsilon \to 0}{\text{lim}}N^{\left(2\right)}/N^{\left(1\right)}=\phi_{12}(\theta )$$

with probability 1 under *H*_*a*_: *θ* ∈ Θ − Θ_0_, which indicates that the Bahadur efficiency ratio *ϕ*_12_(*θ*) gives the limiting ratio of sample sizes required by the two statistics to attain an equally small significance level. As a result, $\left\{T_{n}{}^{\left(1\right)}\right\}$ is deemed superior to, i.e. more Bahadur efficient than, $\left\{T_{n}{}^{\left(2\right)}\right\}$ if *ϕ*_12_(*θ*) ≥ 1 under *H*_*a*_: *θ* ∈ Θ − Θ_0_.

#### Bahadur Efficiency for Lancaster Procedure, Weighted Z-test, and Good's test

Consider *m* sequences of test statistics, $\{T_{n_{i}}^{\left(i\right)}\},\;i=1,\;2,\;\cdots ,\;m$ and the corresponding significance levels,$\left\{P_{n_{i}}^{\left(i\right)}\right\}$, where *n*_*i*_ is the sample size for the *i*^*th*^ test statistic. Assume that for each *i* = 1, 2,⋯, *m*, the sequence $\left\{T_{n_{i}}^{\left(i\right)}\right\}$ has a Bahadur efficiency slope *c*_*i*_(*θ*). That is, $\text{Pr}\left(\underset{n_{i}\to \infty }{\text{lim}}-(2/n_{i})\text{ln}P_{n_{i}}^{\left(i\right)}=c_{i}(\theta )\right)=1$ for some *c*_*i*_(*θ*) > 0 under *H*_*a*_: *θ* ∈ Θ − Θ_0_. Assume also that the sample sizes *n*_1_, ⋯, *n*_*m*_ have an average sample size *n* = (*n*_1_ +⋯+*n*_*m*_) / *m* and $\underset{n\to \infty }{\text{lim}}n_{i}/n=\lambda_{i}$ for *i* = 1, 2,⋯, *m*. Then we have $\text{Pr}\left(\underset{n\to \infty }{\text{lim}}-(2/n)\text{ln}P_{n_{i}}^{\left(i\right)}=\lambda_{i}c_{i}(\theta )\right)=1$. For each *n*, it is desired to combine the *m* statistics $T_{n_{1}}^{\left(1\right)},\;\cdots ,\;T_{n_{m}}^{\left(m\right)}$ into an overall test statistic *T*_*n*_ for testing *H*_0_: *θ* ∈ Θ_0_ vs. *H*_*a*_: *θ* ∈ Θ − Θ_0_.

We first derive the Bahadur efficiency for the Lancaster test. Let *f*_*i*_, *F*_*i*_ and $F_{i}^{-1}$ be the PDF, CDF, and inverse CDF for $\chi_{w_{i}}^{2}$, with *w*_*i*_ > 0 for *i* = 1, 2,⋯, *m*.

**[Theorem 1]** Assume *m* independent test statistics $T_{n_{1}}^{\left(1\right)},\;\cdots ,\;T_{n_{m}}^{\left(m\right)}$ have significance levels $P_{n_{1}}^{\left(1\right)},\;\cdots ,\;P_{n_{m}}^{\left(m\right)}$ respectively. The Lancaster statistic, $\sum_{i=1}^{m}F_{i}^{-1}(1-P_{n_{i}}^{\left(i\right)})$, has the Bahadur efficiency slope $c_{Lancaster}(\theta )=\sum_{i=1}^{m}\lambda_{i}c_{i}(\theta )$ under *H*_*a*_: *θ* ∈ Θ − Θ_0_.

[13] derived the Bahadur efficiency slope for the regular (unweighted) Z-test, $\sum_{i=1}^{m}\Phi^{-1}(1-P_{n_{i}}^{\left(i\right)})/\sqrt{m}$. Here we generalize their findings to the weighted Z-test.

**[Theorem 2]** Assume *m* independent test statistics $T_{n_{1}}^{\left(1\right)},\;\cdots ,\;T_{n_{m}}^{\left(m\right)}$ have significance levels $P_{n_{1}}^{\left(1\right)},\;\cdots ,\;P_{n_{m}}^{\left(m\right)}$ respectively. The weighted Z-test, $\sum_{i}w_{i}\Phi^{-1}(1-P_{n_{i}}^{\left(i\right)})/\sqrt{\sum_{i}w_{i}}$, has the Bahadur efficiency slope $c_{Weighted\;z}(\theta )={\left(\sum_{i=1}^{m}w_{i}\sqrt{\lambda_{i}c_{i}(\theta )}/\sqrt{\sum_{i=1}^{m}w_{i}^{2}}\right)}^{2}$ under *H*_*a*_: *θ* ∈ Θ − Θ_0_.

When *w*_*i*_ = 1 for all *i* = 1, 2,⋯, *m*, the weighted Z-test is reduced to the regular z statistic and the Bahadur efficiency slope is $c_{regular\;z}(\theta )={\left(\sum_{i=1}^{m}\sqrt{\lambda_{i}c_{i}(\theta )}/\sqrt{m}\right)}^{2}$. This finding is in agreement with the Bahadur efficiency finding for the regular Z-test in [13].

The Lancaster test statistic is superior to the weighted Z-test and regular Z-test in terms of Bahadur efficiency. Using the induction method, we show that the Bahadur relative efficiency *ϕ*_12_ = *c*_*Lancaster*_ (*θ*)/ *c*_*Weighted z*_ (*θ*) ≥ 1 and *ϕ*_12_ = *c*_*Lancaster*_ (*θ*)/ *c*_*regular z*_ (*θ*) ≥ 1 for all *θ* ∈ Θ − Θ_0_. The fact that $\underset{\varepsilon \to 0}{\text{lim}}N^{\left(2\right)}/N^{\left(1\right)}=\phi_{12}(\theta )$ indicates that the Lancaster procedure will require smaller sample sizes as compared to the weighted Z-test to achieve the same significance level.

[17] suggested a weighted Fisher’s method. Let $Q=\overset{m}{\underset{i=1}{\text{Π}}}{\left(P_{n_{i}}^{\left(i\right)}\right)}^{w_{i}}$, so $-\text{ln}(Q)=-\sum_{i=1}^{m}w_{i}\text{ln}(P_{n_{i}}^{\left(i\right)})$. When the weights are unequal, the null CDF of *Q* is given by $\text{Pr}(Q<q)=\sum_{i=1}^{m}{\text{Λ}}_{i}q^{1/w_{i}},\;q\in [0,\;1]$, where ${\text{Λ}}_{i}={\left(w_{i}\right)}^{m-1}/\underset{j\neq i}{\text{Π}}\left(w_{i}-w_{j}\right)$. Below we will derive the Bahadur efficiency for Good’s test.

**[Theorem 3]** Assume *m* independent test statistics $T_{n_{1}}^{\left(1\right)},\;\cdots ,\;T_{n_{m}}^{\left(m\right)}$ have significance levels $P_{n_{1}}^{\left(1\right)},\;\cdots ,\;P_{n_{m}}^{\left(m\right)}$ respectively. Good’s test statistic, $-\text{ln}(Q)=-\sum_{i=1}^{m}w_{i}\text{ln}(P_{n_{i}}^{\left(i\right)})$, has the Bahadur efficiency slope $c_{Good}(\theta )=\sum_{i=1}^{m}w_{i}\lambda_{i}c_{i}(\theta )/\underset{i}{\text{max}}(w_{i})$ under *H*_*a*_: *θ* ∈ Θ − Θ_0_.

The maximal weight, $\underset{i}{\text{max}}(w_{i})$, has a strong impact on the Bahadur efficiency in Good’s test. Only the individual test(s) assigned with the maximal weight reserves its Bahadur efficiency in Good's test. That is, if $w_{i}=\underset{i}{\text{max}}(w_{i})$, then $w_{i}\lambda_{i}c_{i}(\theta )/\underset{i}{\text{max}}(w_{i})=\underset{i}{\text{max}}(w_{i})\lambda_{i}c_{i}(\theta )/\underset{i}{\text{max}}(w_{i})=\lambda_{i}c_{i}(\theta )$. Other individual tests will relatively lose more Bahadur efficiency as the maximal weight gets larger. That is, if $w_{i}<\underset{i}{\text{max}}(w_{i})$, then $w_{i}\lambda_{i}c_{i}(\theta )/\underset{i}{\text{max}}(w_{i})<\lambda_{i}c_{i}(\theta )$.

The Lancaster procedure is superior to Good’s test in terms of the Bahadur efficiency, i.e. *ϕ*_12_ = *c*_*Lancaster*_ (*θ*)/ *c*_*Good*_ (*θ*) ≥ 1 for all *θ* ∈ Θ − Θ_0_ and $\underset{\varepsilon \to 0}{\text{lim}}N^{\left(2\right)}/N^{\left(1\right)}=\phi_{12}(\theta )$. For large-scale tests, which often occur in next-generation sequencing data, the Lancaster procedure will require relatively smaller sample sizes as compared to Good’s test, i.e., *N*^*Lancaster*^ ≤ *N*^*Good*^ when the significance level goes to 0, which represents sparse signaling in high throughput data.

#### Lancaster Procedure Has the Optimal Bahadur Efficiency

We can further prove that the Lancaster procedure reaches the upper bounds of Bahadur efficiency among all non-decreasing *T*_*n*_. Thus the Lancaster procedure has the optimal Bahadur efficiency compared to all other combination methods under mild conditions.

**[Proposition 1]** Let *T*_*n*_ be any function of *m* independent test statistics $T_{n_{1}}^{\left(1\right)},\;\cdots ,\;T_{n_{m}}^{\left(m\right)}$. Let *c*_*any*_ (*θ*) > 0 be the Bahadur efficiency slope of *T*_*n*_. Assume *T*_*n*_ is non-decreasing in a way that $t_{1}\leq t_{1}^{*},\;\cdots ,\;t_{m}\leq t_{m}^{*}\;\Rightarrow \;T_{n}(t_{1},\;\cdots ,\;t_{m})\leq T_{n}(t_{1}^{*},\;\cdots ,\;t_{m}^{*})$. Then the Lancaster statistics have the optimal Bahadur efficiency, with *c*_*Lancaster*_ (*θ*) ≥ *c*_*any*_ (*θ*) for all *θ* ∈ Θ − Θ_0_.

The Lancaster procedure and Fisher’s test both have the optimal Bahadur efficiency among all non-decreasing combined tests. Since the Lancaster procedure can incorporate weight functions for auxiliary information in modeling and testing, the Lancaster procedure has more flexibility and it can be considered as the optimal generalized Fisher’s method. The non-decreasing condition, $t_{1}\leq t_{1}^{*},\;\cdots ,\;t_{m}\leq t_{m}^{*}\;\Rightarrow \;T_{n}(t_{1},\;\cdots ,\;t_{m})\leq T_{n}(t_{1}^{*},\;\cdots ,\;t_{m}^{*})$, is easy to meet in practice.

#### Comparing Bahadur Efficiency for Correlated Data

It is critical to assess Bahadur efficiency for correlated data as it will shed light on the impact of correlation structures on the asymptotic convergence rate of significance levels and will further impact the sample sizes required for the experiments. This is a challenging topic since the distributions of combined test statistics under complex correlation structures have no closed analytical forms. To address this issue, we give an approximate Bahadur efficiency using the techniques described in the Methods Section. Below are some interesting results.

**[Proposition 2]** When *m* statistics $T_{n_{1}}^{\left(1\right)},\;\cdots ,\;T_{n_{m}}^{\left(m\right)}$ are correlated, under *H*_*a*_: *θ* ∈ Θ − Θ_0_:

- the Lancaster statistic, has an approximate Bahadur efficiency slope
  $$c_{Lancaster}^{Correlated}(\theta )\approx \frac{\sum_{i}w_{i}}{\sum_{i}w_{i}+2\sum_{i<j}\rho_{ij}}\sum_{i=1}^{m}\lambda_{i}c_{i}(\theta ),$$
  where
  $$\rho_{ij}=\text{cov}\left(F_{i}^{-1}(1-P_{n_{i}}^{\left(i\right)}),F_{j}^{-1}(1-P_{n_{j}}^{\left(j\right)})\right);$$
- the Good’s test statistic has an approximate Bahadur efficiency slope
  $$c_{Good}^{Correlated}(\theta )\approx \frac{\sum_{i}w_{i}}{\sum_{i}w_{i}^{2}+2\sum_{i<j}{\tilde{\rho }}_{ij}}\sum_{i=1}^{m}w_{i}\lambda_{i}c_{i}(\theta ),$$
  where
  $${\tilde{\rho }}_{ij}=\text{cov}\left(\text{ln}(P_{n_{i}}^{\left(i\right)}),\text{ln}(P_{n_{j}}^{\left(j\right)})\right);$$
- the Fisher’s statistic has an approximate Bahadur efficiency slope
  $$c_{Fisher}^{Correlated}(\theta )\approx \frac{m}{m+2\sum_{i<j}{\tilde{\rho }}_{ij}}\sum_{i=1}^{m}\lambda_{i}c_{i}(\theta ).$$

## Simulation Study

We conducted an extensive simulation study to assess the type I error and power of the SKAT-Lancaster procedure. We further compared our proposed method to Gene Set Enrichment Analysis (GSEA) [5]. The empirical assessment was based on rigorous simulation algorithms for sequencing-based genome-wide association studies [18].

The simulation was conducted using the whole exome sequencing genotype data from the 1000 Genomes Project Phase 1 study (n = 822 individuals). After filtration, 40,918 biallellic protein-changing coding variants in selected pathways were mapped to KEGG and Biocarta pathways. To avoid testing over- or under- sized pathways, we selected pathways containing 10 to 100 genes. This yielded 353 pathways with 3304 genes for our simulation study.

We applied a genome-wide additive model to evaluate pathway-testing methods using realistic genetic architectures. Let *Y*_*i*_ = *X*_*i*_*β* + *ε*_*i*_, where *Y*_*i*_ is a continuous trait, the vector *X*_*i*_ is the whole exome sequencing genotype for the *i*^*th*^ subject and $\varepsilon_{i}\overset{i.i.d.}{\sim }N(0,\;\sigma^{2})$ is random noise. The vector *β* contains genetic effect regression coefficients corresponding to genotype variants. In simulation, the *j*^*th*^ variant is causal if |*β*_*j*_| > 0; pathways and genes are causal if they harbor causal variants. We adopted a stochastic hierarchical effect model *β*_*pgv*_ = *C*_*p*_ × *C*_*g*_*d*_*g*_ × *C*_*gv*_*d*_*gv*_ × *e*_*pgv*_ to distribute the total genetic variance into pathways, genes, and individual variants [18]. Within a central causal pathway, we first randomly selected 50% of the genes to be associated with the trait. Then we randomly selected 70% of the variants in causal genes to be associated with the trait. We randomly assigned 80% (20%) of causal genes to be detrimental (protective). For variants within causal genes, 80% were detrimental and 20% were protective. We set the whole-genome heritability *h*_2_ = *Var*(*Xβ*)/(*Var*(*Xβ*) + *σ*^2^) = 20%. This resembles heritability in real data, which often ranges between 20% and 30%. We used Bonferroni correction to control Family-Wise Error Rate (FWER) and set the genome-wide significance level at α = 0.05/353 = 1.4164E-4 for testing 353 pathways. We performed principal component analysis and included the top 3 principal components as covariates in regression analyses to adjust for the population stratification.

In the SKAT-Lancaster procedure, we first performed SKAT to test overall effects on the gene level. Then we considered 4 weight functions in the Lancaster procedure when combining p-values among genes in a pathway:

- $\text{Gene size weight }=2\sqrt{\tilde{n}/n_{i}}$, where *n*_*i*_ is the number of SNPs in the *i*^*th*^ gene and $\tilde{n}=median(n_{i})$ is the median gene size. This weight can remove bias when testing overly small or overly generalized pathways.
- AIC weight, BIC weight: these weight functions calculate the degrees of variations summarized by the gene level multi-SNP regression.
- Uniform weight = 2.

**Fig 1:**
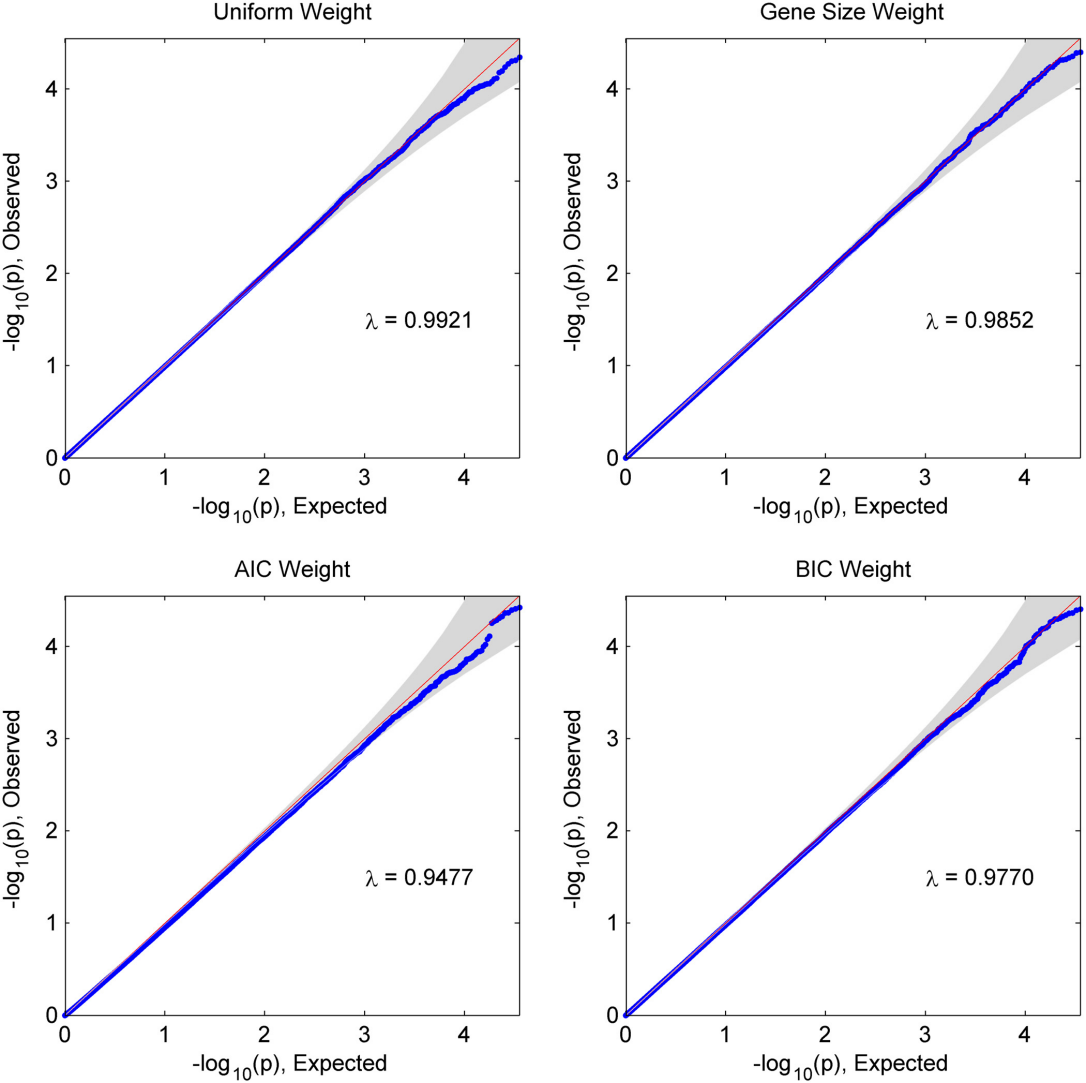
Q-Q plots investigating global null hypothesis type-I errors for the SKAT-Lancaster procedure under Simulation Scenario 1 (*λ* is the inflation factor for the Type I error rate). The type I error inflation factor (*λ*) is the ratio between the area under the curve and the area under the diagonal reference line.

We considered 3 simulation scenarios (Table 1). In Scenario 1, we assessed the global null hypothesis type I error by setting all genetic effect coefficients as zero, i.e. $\beta =\vec{0}$. Any pathways or genes reaching the significance level were considered as false positives. The results in Table 2 indicate that the SKAT-Lancaster procedure has well-controlled type I error rates (~10E-4). We further investigated the Q-Q plot by comparing observed p-values versus expected p-values (Fig 1). The type I error inflation factor (*λ*) is the ratio between the area under the curve and the area under the diagonal reference line. Fig 1 indicates that SKAT-Lancaster procedure with 4 weight functions has no inflation of the global null hypothesis type I error rate (*λ* < 1).

**Table 1:** Simulation Scenarios and parameters*.

Simulation Scenarios 1
Include 353 pathways, 3304 genes.
Phenotype is normally distributed.
Assume heritability is 20%.
$\beta =\vec{0}$.
No pathways, genes or variations are associated with the trait.
Significance level is 0.05/353. All significant results are considered as type 1 errors.
Simulation Scenario 2
Include 353 pathways and 3304 genes.
Phenotype is normally distributed.
Randomly assign one central causal pathway. Within the central causal pathway, randomly assign 50% causal genes. Randomly assign 70% causal variants in associated genes.
Randomly assigned 80% (20%) of causal genes to be detrimental (protective). For variants within the causal genes, 80% are detrimental and 20% are protective.
Associated variants' effect size ~ log_10_(*MAF*).
Significance level is 0.05/353.
Simulation Scenarios 3
Include 353 pathways and 3304 genes.
Phenotype is normally distributed.
Assume heritability is 20%.
Randomly assign one central causal pathway. Within the central causal pathway, randomly assign 50% causal genes. Randomly assign 70% causal variants in associated genes.
Randomly assigned 80% (20%) of causal genes to be detrimental (protective). For variants within the causal genes, 80% are detrimental and 20% are protective.
Associated variants' effect size ~$1/\sqrt{MAF*(1-MAF)}$.
Significance level is 0.05/353.

* Covariates: top 3 principal components for population stratification are included as covariates in all three simulation scenarios.

**Table 2:** Comparison of type I error and power among competing methods.

Simulation Scenario 1			
		Type_1 error	Inflation factor
Test	*Weight function*	(10E-4)	*λ*
SKAT- Lancaster	*Uniform*	1.1615	0.9921
SKAT- Lancaster	*Gene size*	1.3598	0.9852
SKAT- Lancaster	*AIC*	0.9632	0.9477
SKAT- Lancaster	*BIC*	1.1331	0.9770
GSEA		12.0000	1.2390
Simulation Scenario 2			
Test	*Weight function*	Stringent Power	Lenient Power
SKAT- Lancaster	*Uniform*	0.870	0.884
SKAT- Lancaster	*Gene size*	0.810	0.836
SKAT- Lancaster	*AIC*	0.832	0.854
SKAT- Lancaster	*BIC*	0.809	0.826
GSEA		0.279	0.373
Simulation Scenario 3			
Test	*Weight function*	Stringent Power	Lenient Power
SKAT- Lancaster	*Uniform*	0.610	0.645
SKAT- Lancaster	*Gene size*	0.509	0.543
SKAT- Lancaster	*AIC*	0.585	0.628
SKAT- Lancaster	*BIC*	0.540	0.558
GSEA		0.468	0.505

In Scenarios 2 and 3, we assessed the stringent power and lenient power when randomly generating one central causal pathway in each simulation (Table 1). The stringent power calculates the percentage of times the central causal pathway is found significant. Due to the correlation among pathways, pathways that share causal genes with the central causal pathway are overlapping causal pathways. The lenient power calculates the percentage of times (central and overlapping) causal pathways are found significant.

The results in Table 2 indicate that the SKAT-Lancaster procedure outperformed GSEA. In Scenario 2, the SKAT-Lancaster procedure with 4 weight functions had lenient power ranging between 0.826 and 0.884, while GSEA had lenient power of 0.373. In Scenario 3, the SKAT-Lancaster procedure with 4 weight functions had lenient power ranging between 0.543 and 0.645, while GSEA had lenient power of 0.505. We randomly assign the causal variances in Scenarios 2 and 3, the SKAT-Lancaster procedure with uniform weight had the best detection power.

Regarding the computing time, the self-contained Lancaster procedure compares a test statistic to an asymptotic distribution, thus it does not require intensive computation. The competitive Lancaster procedure is based on permutation and it has similar computation efficiency as compared with GSEA.

## Case Study: Lipid Meta-Analysis

We illustrate our method using meta-analysis data generated by the Global Lipids Genetics Consortium. To identify new loci and validate existing loci associated with lipids, [19] we analyzed the levels of low-density lipoprotein (LDL) cholesterol, high-density lipoprotein (HDL) cholesterol, triglycerides (TG) and total cholesterol (TC) of 196,475 individuals from 60 studies. A total of 1,048,161 Single Nucleotide Polymorphisms (SNPs) were genotyped using the genome-wide association study (GWAS) arrays and Metabochip arrays. These variants were selected from promising loci associated with lipid and coronary artery disease, based on findings from previous GWAS studies and the 1000 Genome Project. Subjects taking lipid-lowering medications were excluded in the meta-analysis. The additive effect of each SNP on blood lipid levels after adjusting for age and sex was analyzed and p-value was generated for each SNP and each lipid variable. Genomic control values for the initial meta-analyses were 1.10–1.15, indicating that population stratification had only a minor impact on the results [20].

The SKAT-Lancaster procedure can only be applied to original data. Remarkably, as the Lancaster procedure is independent from the SKAT test, it can be applied to secondary data analysis. To identify pathways that are more significant than others, we performed the competitive Lancaster procedure. In the competitive test, we performed 100,000 times of permutations and ensured that the permutated pathways preserved the size and characteristics of original pathways. Our simulation study showed that the competitive Lancaster procedure had well-controlled type I error rates to prevent false discoveries.

Before comparing the proposed method to Fisher’s method [21] and weighted Z-test [22], we considered 4 weight functions for the Lancaster procedure:

- $w_{1}=2\sqrt{\tilde{n}/n_{i}}$, where *n*_*i*_ is the number of SNPs in the *i*^*th*^ gene and $\tilde{n}=median(n_{i})$ is the median gene size. This is a weight adjusted by gene size to remove the bias from large genes.
- $w_{2}=4\sqrt{MAF(1-MAF)}$, where MAF stands for minor allele frequency. Common variants receive higher weights.
- $w_{3}=1/\sqrt{MAF(1-MAF)}$. Rare variants receive higher weights.
- Uniform weight: *w*_4_ = 2.

**Fig 2:**
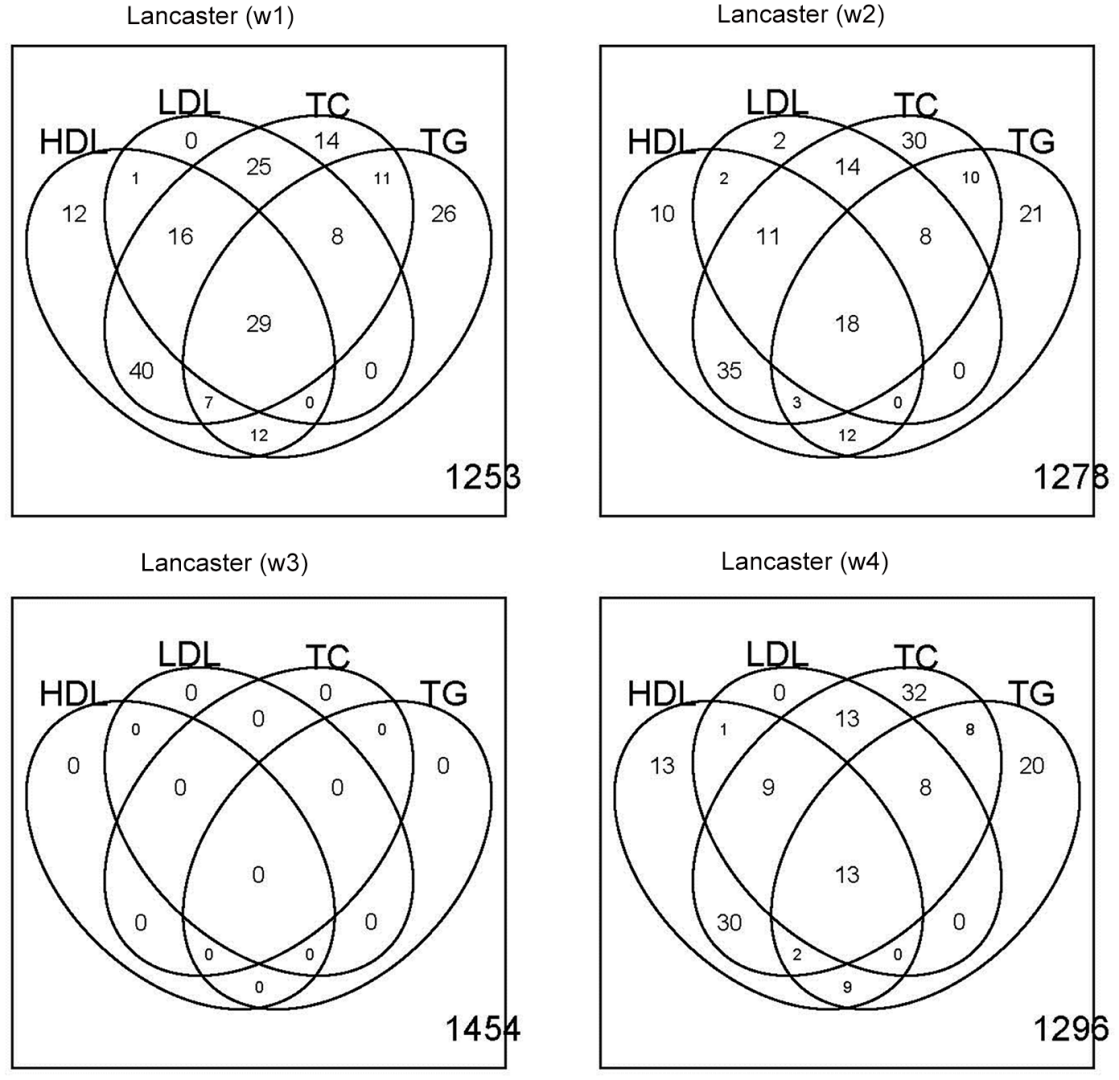
Venn Diagrams for Significant Pathways (FDR < 0.05).

Pathway analysis was performed using the gene ontology (GO) gene sets from http://www.broadinstitute.org/gsea/index.jsp. A total of 1454 pathways were analyzed and multiple testing was adjusted by False Discovery Rate (FDR) [23]. The numbers of significant pathways are summarized in Fig 2 As shown in Table 3, the Lancaster procedure outperformed Fisher's method and weighted Z-test by identifying more significant pathways. When the Lancaster procedure was assigned with uniform weights (*w*_4_), it performed equivalently to Fisher's method. The weighted Z-test is not optimal in Bahadur efficiency, so it identified fewer pathways than the Lancaster procedure and Fisher's method. Weight functions *w*_1_ and *w*_2_ outperformed *w*_3_ and *w*_4_, indicating that removing gene size bias and assigning higher weights to common variants can improve power of the Lancaster procedure.

**Table 3:** Number of Significant pathways.

FDR < 0.05	HDL	LDL	TC	TG
Lancaster (w1)	117	79	150	93
Lancaster (w2)	91	55	129	72
Lancaster (w3)	0	0	0	0
Lancaster (w4)	77	44	115	60
Fisher	77	44	115	60
Weighted Z-test (w1)	2	1	3	2
Weighted Z-test (w2)	5	0	5	4
Weighted Z-test (w3)	0	0	0	0
Weighted Z-test (w4)	4	0	4	6

We compared our pathway findings with findings from the MAGENTA analysis in [19] (Table 4). The Lancaster procedure (*w*_1_) showed that the "enzyme binding" pathway is significantly associated with HDL (FDR<10^−5^), which agrees with the finding from [19] (FDR = 0.038). The "enzyme binding" pathway contained 178 genes interacting selectively and non-covalently with any enzyme. The Lancaster procedure (*w*_1_, *w*_2_, *w*_4_) showed that the "lipid transport" pathway is significantly associated with LDL (FDR adjusted p-value <10^−5^), which agrees with the finding from [19] (FDR = 0.0016). The "lipid transport" pathway contains 28 genes involving directed movement of lipids into, out of, within, or between cells. Lipids are compounds soluble in an organic solvent but not, or sparingly, in an aqueous solvent. The Lancaster procedure (*w*_1_, *w*_2_, *w*_4_)found that the "lipoprotein metabolic process" pathway is significantly associated with LDL (FDR adjusted p-value <10^−5^), which agrees with the finding from [19] (FDR = 0.00017). The "lipoprotein metabolic process" pathway contains 33 genes involving the chemical reactions. The pathway also involves any conjugated, water-soluble protein in which the non-protein moiety consists of a lipid or lipids.

**Table 4:** Comparison of pathway analysis p-values.

Pathway Name	enzyme binding	lipid transport	lipoprotein metabolic process
GO Accession	GO:0019899	GO:0006869	GO:0042157
Gene Ontology	molecular function	biological process	biological process
Description	Interacting selectively with any enzyme	The directed movement of lipids into, out of, within or between cells. Lipids are compounds soluble in an organic solvent but not, or sparingly, in an aqueous solvent.	The chemical reactions and pathways involving any conjugated, water-soluble protein in which the nonprotein moiety consists of a lipid or lipids.
number of genes	178	28	33
number of SNPs	12089	1058	769
(Willer 2013)*	0.038	0.0016	0.00017
Lancaster (w1)*	<10^−5^	<10^−5^	<10^−5^
Lancaster (w2) *	0.80	<10^−5^	<10^−5^
Lancaster (w3)*	0.98	0.44	0.44
Lancaster (w4)*	0.82	<10^−5^	<10^−5^
Fisher*	0.82	<10^−5^	<10^−5^
Weighted z (w1)*	0.90	0.36	0.58
Weighted z (w2)*	0.94	0.21	0.35
Weighted z (w3)*	0.99	0.65	0.55
Weighted z (w4)*	0.94	0.23	0.36

* FDR adjusted p-values

## Discussion and Conclusions

The proposed two-stage approach is a powerful tool to integrate information in pathway analysis of sequencing association studies. The first stage is the gene-based testing, where effects from rare variants within a gene are summarized into one p-value using the SKAT test. In the second stage, p-values from multiple genes are combined for pathway analysis and meta-analysis using the correlated Lancaster procedure. In this work, we prove that the Lancaster procedure is optimal in Bahadur efficiency among all combined p-value methods.

We assess the Bahadur efficiency among weighted combined p-value methods and further prove that the Lancaster procedure is optimal in Bahadur efficiency under very mild conditions. There has been a lack of theatrical comparison among combined p-value methods. Several simulation studies have compared weighted combined p-value methods [15, 22, 24]. With more than 400 citations in the literature, these studies have been a subject of intense interest to the research community heated discussions in the research community, but yield controversial results in different simulation scenarios. Thus, we fill the gap by comparing the Bahadur efficiency among methods.

The Bahadur efficiency is a critical measure of performance of statistical testing [25] [26]. In [25], Bahadur efficiency has been applied for sensitivity analyses in observation studies. The Bahadur efficiency, $\underset{\varepsilon \to 0}{\text{lim}}N^{\left(2\right)}/N^{\left(1\right)}=\phi_{12}(\theta )$, compares sample sizes among different statistical tests when signals become sparse in sequencing data, i.e. *ε* → 0. As the number of genetic variants scanned by the sequencing technology increases from thousands to millions, signals that are associated with phenotypes become sparse, requiring a more stringent statistical significance level to detect sparse signals, i.e. ($P_{N^{\left(i\right)}}<\varepsilon \to 0$). The optimal Bahadur efficiency ensures that the Lancaster procedure asymptotically requires a minimal sample size to detect sparse signals.

Among combined p-value methods, the Lancaster procedure can be considered as the generalized Fisher's method with a weight function. Weight functions, when used appropriately, can generally increase the power of combined p-value methods [27–29].

Evaluating Bahadur efficiency for high-throughput genetic data is critical since there is no combined p-value method that that is uniformly the most powerful. Bahadur efficiency calculates the limiting ratio of sample sizes required by two statistics to attain an equally small significance level. The optimal Bahadur efficiency indicates that the Lancaster procedure asymptotically requires a minimal sample size to attain the significance level.

### Data and Software

R package ‘CombinePValue’ has been created for the proposed Lancaster procedure. Case study data are available from http://csg.sph.umich.edu//abecasis/public/lipids2013/. Source codes for simulation analyses can be provided upon contacting Dr Guodong Wu.

## Appendix

Lemma 1 [16, 30] is needed to derive the Bahadur efficiency.

**[Lemma 1]** If the following two conditions are met,

(Condition 1) there exists a function *b*(*θ*), 0 < *b*(*θ*) < ∞, such that $T_{n}/\sqrt{n}\to b(\theta )$ with probability 1 under *H*_*a*_: *θ* ∈ Θ − Θ_0_;
(Condition 2) there exists a function *f*(*t*), 0 < *f*(*t*) < ∞, which is continuous in some open set containing the range of *b*(*θ*) such that for each *t* in the open set $-n^{-1}\text{ln}\left[1-F_{0}\left(\sqrt{n}t\right)\right]\to f(t)$, then the Bahadur efficiency slope of {*T*_*n*_} is c(*θ*) = 2*f* (*b*(*θ*)).

**Proof of Theorem 1:** Since the equivalent tests have the same Bahadur efficiency, we can consider $T_{n}^{Lancaster}=\sqrt{\sum_{i=1}^{m}Z_{n_{i}}^{\left(i\right)}}$, where $Z_{n_{i}}^{\left(i\right)}=F_{i}^{-1}(1-P_{n_{i}}^{\left(i\right)})$. Under *H*_*a*_: *θ* ∈ Θ_0_, $P_{n_{i}}^{\left(i\right)}\sim Uniform(0,\;1)$ and $Z_{n_{i}}^{\left(i\right)}\sim \chi_{w_{i}}^{2}$. According to Theorem 2.1 by [31], we have $-2\text{ln}P_{n_{i}}^{\left(i\right)}=-2\text{ln}(1-F_{i}(Z_{n_{i}}^{\left(i\right)}))=-2\text{ln}(f_{i}(Z_{n_{i}}^{\left(i\right)}))+o(1)=Z_{n_{i}}^{\left(i\right)}(1+o(1))$ as $Z_{n_{i}}^{\left(i\right)}\to \infty$. So $n^{-1}Z_{n_{i}}^{\left(i\right)}(1+o(1))=-(2/n)\text{log}P_{n_{i}}^{\left(i\right)}\to \lambda_{i}c_{i}(\theta )$ with probability 1 under *H*_*a*_: *θ* ∈ Θ − Θ_0_. It follows that

$$T_{n}^{Lancaster}/\sqrt{n}=\sqrt{\sum_{i=1}^{m}Z_{n_{i}}^{\left(i\right)}/n}\to \sqrt{\sum_{i=1}^{m}\lambda_{i}c_{i}(\theta )}.$$ (1)

Now, for *θ* ∈ Θ_0_, $T_{n}^{Lancaster}$ is distributed as the square root of $\chi_{\sum_{i}w_{i}}^{2}$ with the CDF *F* and PDF *f*. According to Theorem 2.1 by [31], we have,

$$-n^{-1}\text{ln}\left[1-F\left(\sqrt{n}t\right)\right]=-n^{-1}\text{ln}\left[f\left(\sqrt{n}t\right)\right]=0.5t^{2}(1+o(1))\to 0.5t^{2}.$$ (2)

Plug the results from Eqs (1) and (2) to Lemma 1. Then the Bahahur efficiency slope for the Lancaster statistic is $c_{Lancaster}(\theta )=\sum_{i=1}^{m}\lambda_{i}c_{i}(\theta )$ under *H*_*a*_: *θ* ∈ Θ − Θ_0_.

**Proof of Theorem 2:** Rewrite the weighted z statistic as $T_{n}^{Weighted\;z}=\sum_{i=1}^{m}(w_{i}Z_{n_{i}}^{\left(i\right)})/\sqrt{\sum_{i=1}^{m}w_{i}^{2}}$, where $Z_{n_{i}}^{\left(i\right)}=\Phi^{-1}(1-P_{n_{i}}^{\left(i\right)})$. Under *H*_0_: *θ* ∈ Θ_0_, $P_{n_{i}}^{\left(i\right)}\sim Uniform(0,\;1)$ and $Z_{n_{i}}^{\left(i\right)}\sim N(0,\;1)$. According to Theorem 2.1 by [31], we have $-2\text{ln}P_{n_{i}}^{\left(i\right)}=-2\text{ln}(1-\Phi (Z_{n_{i}}^{\left(i\right)}))=-2\text{ln}(f(Z_{n_{i}}^{\left(i\right)}))+o(1)={\left[Z_{n_{i}}^{\left(i\right)}\right]}^{2}(1+o(1))$ as $Z_{n_{i}}^{\left(i\right)}\to \infty$. So $n^{-1}{\left[Z_{n_{i}}^{\left(i\right)}\right]}^{2}(1+o(1))=-2n^{-1}\text{log}P_{n_{i}}^{\left(i\right)}\to \lambda_{i}c_{i}(\theta )$ with probability 1 under *H*_*a*_: *θ* ∈ Θ − Θ_0_. It follows that

$$T_{n}^{Weighted\;z}/\sqrt{n}=\sum_{i=1}^{m}(w_{i}Z_{n_{i}}^{\left(i\right)})/\sqrt{n\sum_{i=1}^{m}w_{i}^{2}}\to \sum_{i=1}^{m}w_{i}\sqrt{\lambda_{i}c_{i}(\theta )}/\sqrt{\sum_{i=1}^{m}w_{i}^{2}}.$$ (3)

Now, for *θ* ∈ Θ_0_, $T_{n}^{Weighted\;z}\sim \;N(0,\;1)$. Thus,

$$-n^{-1}\text{ln}\left[1-\text{Φ}\left(\sqrt{n}t\right)\right]=0.5t^{2}(1+o(1))\to 0.5t^{2}.$$ (4)

Eqs (3) and (4) and Lemma 1 imply that the Bahadur efficiency slope of $\left\{T_{n}^{Weighted\;z}\right\}$ is $c_{Weighted\;z}(\theta )={\left(\sum_{i=1}^{m}w_{i}\sqrt{\lambda_{i}c_{i}(\theta )}/\sqrt{\sum_{i=1}^{m}w_{i}^{2}}\right)}^{2}.$

**Proof of Theorem 3:** It is equivalent to consider $T_{n}^{Good}=\sqrt{-\sum_{i=1}^{m}w_{i}\text{ln}(P_{n_{i}}^{\left(i\right)})}$. Then

$$T_{n}^{Good}/\sqrt{n}=\sqrt{-n^{-1}\sum_{i=1}^{m}w_{i}\text{ln}(P_{n_{i}}^{\left(i\right)})}\to \sqrt{0.5\sum_{i=1}^{m}w_{i}\lambda_{i}c_{i}(\theta )}$$

with probability 1 under *H*_*a*_: *θ* ∈ Θ − Θ_0_. Direction calculation shows that the PDF of $\sqrt{-\text{ln}(Q)}$ is $f(x)=2x\sum_{i=1}^{m}\frac{{\text{Λ}}_{i}}{w_{i}}\text{exp}(-x^{2}/w_{i})$ under *H*_0_. Let *I* be the index corresponds to $\underset{i}{\text{max}}(w_{i})=w_{I}$. One can construct the upper and lower bounds of *f*(*x*),

$$2x\frac{{\text{Λ}}_{I}}{w_{I}}\text{exp}(-x^{2}/w_{I})\leq f(x)\leq 2xm\frac{{\text{Λ}}_{I}}{w_{I}}\text{exp}(-x^{2}/w_{I})$$

when *x* is greater than a certain finite number. This implies that

$$-n^{-1}\text{ln}\left[1-F\left(\sqrt{n}t\right)\right]=-n^{-1}\text{ln}f\left(\sqrt{n}t\right)+o(1)\to t^{2}/\text{max}(w_{i}).$$

By Lemma 1, the Bahadur efficiency slope for Good’s test is $c_{Good}(\theta )=\sum_{i=1}^{m}w_{i}\lambda_{i}c_{i}(\theta )/\underset{i}{\text{max}}(w_{i})$ for *θ* ∈ Θ − Θ_0_.

**Proof of Proposition 1:** Let *P*_*n*_ be the significance level of *T*_*n*_ and let *t*^(1)^, ⋯, *t*^(*m*)^ be the observed values of $T_{n_{1}}^{\left(1\right)},\;\cdots ,\;T_{n_{m}}^{\left(m\right)}$. For any non-decreasing *T*_*n*_, we have

$$P_{n}\geq \text{Pr}\left(T_{n_{1}}^{\left(1\right)}>t^{\left(1\right)},\;\cdots ,\;T_{n_{m}}^{\left(m\right)}>t^{\left(m\right)}\right)\geq \overset{m}{\underset{i=1}{\text{Π}}}\text{Pr}\left(T_{n_{i}}^{\left(i\right)}>t^{\left(i\right)}\right).$$

Therefore, $c_{Lancaster}(\theta )=\sum_{i=1}^{m}\lambda_{i}c_{i}(\theta )=-n^{-1}\sum_{i=1}^{m}\text{ln}\left(\text{Pr}(T_{n_{i}}^{\left(i\right)}>t^{\left(i\right)})\right)\geq -n^{-1}\text{ln}(P_{n})=c_{any}(\theta )$ for all *θ* ∈ Θ − Θ_0_.

**Proof of Proposition 2:** We give the proof to the Lancaster statistic. Note that any correlation structure among p-values has no impact to the first condition of Lemma 1. As a result, one can repeat the derivation for Theorem 1 to get

$$T_{n}^{Lancaster}/\sqrt{n}=\sqrt{\sum_{i=1}^{m}Z_{n_{i}}^{\left(i\right)}/n}\to \sqrt{\sum_{i=1}^{m}\lambda_{i}c_{i}(\theta )}$$ (5)

under *H*_*a*_: *θ* ∈ Θ − Θ_0_.

When $P_{n_{1}}^{\left(1\right)},\;\cdots ,\;P_{n_{m}}^{\left(m\right)}$ are correlated, the null distribution of $T_{n}^{Lancaster}=\sum_{i=1}^{m}F_{i}^{-1}(1-P_{n_{i}}^{\left(i\right)})$ no longer follows $\chi_{\sum_{i}w_{i}}^{2}$ distribution. One can approximate it by a scaled chi-square distribution such as $T_{n}^{Lancaster}\approx c\chi_{v}^{2}$. By matching expectation and variance between two sides, one can solve for *c* and *v*. Under *H*_0_: *θ* ∈ Θ_0_, direct calculations show that

$$-n^{-1}\text{ln}\left[1-F\left(\sqrt{n}t\right)\right]\approx -n^{-1}\text{ln}\left[1-\tilde{F}\left(\sqrt{n}t\right)\right]=-n^{-1}\text{ln}\left[\tilde{f}\left(\sqrt{n}t\right)\right]=\frac{0.5t^{2}\sum_{i}w_{i}}{\sum_{i}w_{i}+2\sum_{i<j}\rho_{ij}}(1+o(1))$$ (6)

where *F* is the CDF of $T_{Lancaster}^{Correlated}$ and $\tilde{F}$ ($\tilde{f}$) are the CDF (PDF) of $c^{-1}\chi_{v}^{2}$.

Plug the results from Eqs (5) and (6) to Lemma 1. Then the Bahahur efficiency slope for the Lancaster statistic is $c_{Lancaster}^{Correlated}(\theta )\approx \frac{\sum_{i}w_{i}}{\sum_{i}w_{i}+2\sum_{i<j}\rho_{ij}}\sum_{i=1}^{m}\lambda_{i}c_{i}(\theta )$ under *H*_*a*_: *θ* ∈ Θ − Θ_0_.
